# Endocervical Adenocarcinoma, Gross Examination, and Processing, Including Intraoperative Evaluation: Recommendations From the International Society of Gynecological Pathologists

**DOI:** 10.1097/PGP.0000000000000745

**Published:** 2021-02-09

**Authors:** Carlos Parra-Herran, Anais Malpica, Esther Oliva, Gian Franco Zannoni, Pedro T. Ramirez, Joseph T. Rabban

**Affiliations:** Department of Pathology, Brigham and Women’s Hospital and Harvard Medical School (C.P.H.); Department of Pathology, Massachusetts General Hospital and Harvard Medical School (E.O.), Boston, Massachusetts; Departments of Pathology (A.M.); Gynecologic Oncology and Reproductive Medicine, The University of Texas MD Anderson Cancer Center (P.T.R.), Houston, Texas; Department of Woman, Child and Public Health Sciences, Gynecopathology and Breast Pathology Unit, Catholic University, Rome, Italy (G.F.Z.); Department of Pathology, University of California San Francisco, San Francisco, California (J.T.R.)

**Keywords:** Endocervical adenocarcinoma, LEEP, Cone, Hysterectomy, Lymph node dissection, Specimen processing

## Abstract

The International Society of Gynecological Pathologists (ISGyP) Endocervical Adenocarcinoma Project aims to provide evidence-based guidance for the pathologic evaluation, classification, and reporting of endocervical adenocarcinoma. This review presents the recommendations pertaining to gross evaluation and intraoperative consultation of specimens obtained from patients in the setting of cervical cancer. The recommendations are the product of review of published peer-reviewed evidence, international guidelines and institutional grossing manuals, as well as deliberation within this working group. The discussion presented herein details the approach to the different specimen types encountered in practice: loop electrosurgical excision procedure, cone, trachelectomy, radical hysterectomy, pelvic exenteration, and lymphadenectomy specimens. Guidelines for intraoperative evaluation of trachelectomy and sentinel lymph node specimens are also addressed. Correlation with ISGyP recommendations on cancer staging, which appear as a separate review in this issue, is also included when appropriate. While conceived in the framework of endocervical adenocarcinoma, most of the discussion and recommendations can also be applied to other cervical malignancies.

The accurate pathologic diagnosis, reporting, and staging of endocervical adenocarcinoma is highly dependent on integrating data from the macroscopic evaluation of the specimen. Thus, a standardized, systematic approach to the management of the gross specimen is essential. The recommendations in this review are a product of the International Society of Gynecological Pathologists (ISGyP) Endocervical Adenocarcinoma Project and reflect an integration of published evidence with expert-based recommendations where evidence is not available. As many of the issues and recommendations discussed herein have not been explored systematically, they are largely a product of consensus obtained from review of current institutional grossing manuals and deliberation within this working group. The recommendations address loop electrosurgical excision procedure (LEEP), cone, trachelectomy, hysterectomy, pelvic exenteration and lymphadenectomy specimens for endocervical adenocarcinoma, highlighting issues for intraoperative evaluation when appropriate. The guidelines address specimen orientation, specimen and tumor measurements, specimen dissection, tissue sampling and tissue block preparation. The implications of macroscopic findings on final tumor reporting and tumor staging are briefly discussed but the reader is referred to the accompanying review in this issue on detailed tumor staging recommendations.

## FRAGMENTED LEEP/LOOP EXCISION OF THE TRANSFORMATION ZONE SPECIMENS

LEEP, also known as large loop excision of the transformation zone, is designed to excise the squamocolumnar junction in patients with colposcopically suspected or cytologically suspected/confirmed high-grade squamous intraepithelial lesion 1. In this setting, LEEP is generally recommended over cold knife conization 2,3. The cutting device is a wire loop that simultaneously cuts tissue and cauterizes the surgical site to achieve hemostasis. Advantages of this technique over cold knife conization are that the hemostasis control permits the procedure to be safely conducted in an office setting (whereas cold knife conization requires an intraoperative setting for hemostasis control) and that the tissue margins are cauterized, allowing easy identification microscopically. A disadvantage of LEEP over cold knife conization is that the cautery-induced thermal artifact may obscure microscopic examination of the tissue at the margin.

**FIG. 1:**
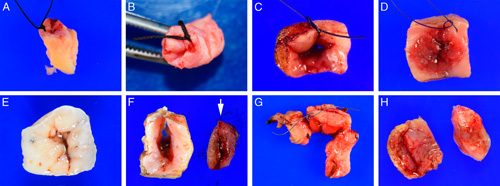
Cold knife cone specimens of the cervix (A–D) may be conical (eg, tapered cylinder) (A), long and cylindrical (B), broad cylinder (C), or cube-like (D), typically oriented by the surgeon with a suture at the 12 o’clock position of the ectocervical mucosa. Loop electrosurgical excision procedure (LEEP) specimens are roughly cylindrical (E) and sometimes may be accompanied by a separate smaller cylinder of endocervix (so-called top hat specimen) that is excised to obtain tissue deeper in the endocervical canal than the main LEEP (F, arrow). Occasionally a cone or LEEP may become disrupted during the surgical procedure (G) or the clinician may need to perform the LEEP as 2 or more separate excisions rather than as a single intact cylinder (H). These 2 latter types of specimens may be challenging to produce well-oriented sections.

Ideally the LEEP procedure is performed in a single circumferential pass with the electrosurgical device around the entire squamocolumnar junction, creating a single intact cylinder of tissue (Figs. 1A–F). In some cases, however, it may not be possible for the surgeon to perform the procedure in a single pass (Figs. 1G, H); instead the surgeon may need to use 2 or more passes with the electrosurgical device to remove the cylindrical target as 2 or more separate pieces of tissue, rather than an intact cylinder. On occasion, the intact cylinder may break open during the procedure and herein referred to as a fragmented LEEP specimen. The term fragmented LEEP does not include the scenario in which an intact cylinder produced by LEEP is accompanied by an additional separate specimen of the more proximal endocervical canal, often referred to as a top-hat (Fig. 1F).

A challenge in evaluating a fragmented LEEP specimen relates to the understanding of the relationship among the different specimens edges and to the true endocervical and ectocervical margins. Thus, specimen management in this scenario is discussed separately from that of an intact LEEP/cone specimens.

### Specimen Orientation

Fragmented LEEP specimens have a mucosal surface on one side and cervical wall connective tissue on the opposite side (so-called deep margin). The ectocervical edge may appear shiny, smooth and white compared to the endocervical edge which may be pink and finely granular with adherent mucus. The deep connective tissue margin is usually rough and cauterized. Recognizing these landmarks is important, when possible, as the ideal tissue slices are cut parallel to the axis of the endocervical canal.

The presence of thermal artifact on microscopic examination distinguishes a true surgical margin from an edge created during the process of slicing or trimming the fragments. Therefore inking the specimen is not required.

#### Recommendations

- The presence of thermal artifact on microscopic examination of the tissue edges of a fragmented LEEP specimen in the best marker of a true surgical margin.

### Specimen and Tumor Measurements

The number of fragments and the range of the dimensions (minimum size and maximum size) should be recorded. The dimensions of any macroscopic lesions should be documented, though the anatomic orientation of the dimensions may be challenging in a fragmented LEEP.

#### Recommendations

- Document the number of tissue fragments and size range (minimum to maximum).

### Specimen Processing and Tissue Sampling

The fragments should be sliced at 2 to 3 mm intervals parallel to the endocervical canal. This will produce a tissue section that exhibits the mucosa along one edge, with the endocervical and ectocervical mucosal margins at either end of this edge. The deep connective tissue margin will be the edge opposite of the mucosal one. All tissue should be submitted for microscopic examination. To mitigate against tangential sectioning artifacts and incomplete visualization of the full mucosa in the hematoxylin and eosin (H&E)-stained fragments, it is recommended to place no more than 1 or 2 tissue sections in each cassette since any more may make it difficult to align all the sections in the same plane, potentially resulting in incomplete representation of the mucosa of all of the sections in the block.

The number of inital H&E-stained sections per block to examine varies between practices though little evidence exists to provide guidance. One study reported that a single H&E section per block was sufficient for accurate diagnosis as long as deeper sections were examined in certain settings after review of the initial H&E, such as missing mucosa, absence of squamous intraepithelial lesion, suspicion for stromal invasion, or findings that are discordant with the clinical, colposcopic, and/or cytologic findings 4. Some professional organizations recommend one initial H&E section per block, with additional sections to be considered depending on the findings in that initial section 5, while many practices evaluate 2 or more initial H&E sections per block in all cases. Further studies are needed to guide best practices.

#### Recommendations

- Slice the tissue in 2 to 3 mm thick slices parallel to the endocervical canal.
- Limit the number of slices per cassette in order to avoid incomplete representation due to sectioning artifacts.
- A single H&E-stained section per block is sufficient for initial microscopic examination, with consideration for deeper sections when there is missing mucosa, absence of squamous intraepithelial lesion/endocervical adenocarcinoma, suspicion for stromal invasion, or findings that are discordant with the clinical, colposcopic, and/or cytologic findings. Alternatively it may be more efficient to routinely examine 2 or more sections per block, depending on the local practice.

## INTACT LEEP AND COLD KNIFE CONE SPECIMENS

Intact LEEP and cold knife cone specimens consist of a cylinder of the endocervical canal with ectocervical mucosa at one end and endocervical mucosa at the other end. The shape and size of the cylinder may vary considerably from one patient to another, with some being long and tapered and other being broad and shallow. The outer surface of the cylinder corresponds to the deep connective tissue margin. Assessment for invasive tumor and for margin involvement by tumor are 2 key goals of the pathologic evaluation. As thermal artifact from the LEEP technique may obscure the microscopic evaluation of the specimen margins, the cold knife cone technique offers better margin assessment since electrocautery is not used. For histologically confirmed endocervical adenocarcinoma in situ, cold knife conization is generally advised over LEEP by some but not all guidelines 2,3.

### Specimen Orientation and Inking

The 3 margins are the mucosal endocervical margin, the mucosal ectocervical margin, and the deep connective tissue margin. If the surgeon has not provided orientation to the endocervical versus ectocervical margin, this can usually be determined by the different appearance of the mucosa. The ectocervical mucosa is shiny, smooth and white when compared with the endocervical mucosa which often is pink and finely granular with adherent mucus. All of the margins should be inked; a single color is sufficient though some pathologists prefer to use 2 colors to further distinguish the endocervical end versus the ectocervical end of the specimen.

The surgeon may designate the anatomic orientation of the cone with a suture, for example indicating the 12 o’clock position or anterior cervical lip. In such a case, the clock face orientation positions should be preserved throughout the specimen sectioning, block designation, and final pathology report. If such orientation is not provided, there is no need for the pathologist to arbitrarily designate clock face orientation positions as there is no way to correlate them with the anatomic landmarks.

#### Recommendations

- Ink the ectocervical and endocervical mucosal margins as well as the deep connective tissue margin.
- If anatomic orientation of the specimen is designated, preserve this orientation in the tissue block designations.

### Specimen Measurements

The length (parallel to the endocervical canal), diameter and wall thickness of the specimen should be recorded. The dimensions of any macroscopically visible tumor should be recorded using the same strategy described for trachelectomy specimens (see below).

#### Recommendations

- Document length, diameter and wall thickness of the specimen.

### Tumor Location

If the surgeon provided orientation of the specimen, then the anatomic location of grossly visible tumor should be recorded as this may assist to correlate with radiologic and intraoperative findings, particularly if there is clinical concern for margin involvement by tumor. Options include using a positions on a clock or designating anterior versus posterior lip of the cervix.

#### Recommendations

- Document the anatomic location of tumor in the cervix using positions on a clock or designating anterior versus posterior lip of the cervix.
- Document the distance of tumor to the endocervical margin, ectocervical margin and deep connective tissue margin.

### Macroscopic Tumor Dimensions

The 3 macroscopic dimensions of a cervical tumor are its length (parallel to the endocervical canal), width (in the plane perpendicular to the endocervical canal), and thickness (from tumor surface to the tumor deepest invasion point). The macroscopic depth of invasion is defined as the distance from the endocervical mucosa to the tumor deepest point within the cervical wall. Depending on the relative amount of exophytic growth versus growth into the cervical wall, tumor thickness and tumor depth of invasion may be different.

#### Recommendations

- Document the tumor length (parallel to the endocervical canal), tumor width (perpendicular to the endocervical canal), tumor thickness, and depth of tumor invasion.

### Specimen Processing and Tissue Sampling

Some intact LEEP/cone specimens may be submitted to the pathology laboratory in the fresh state while others are submitted already in formalin. This affects the strategy for specimen handling.

**FIG. 2:**
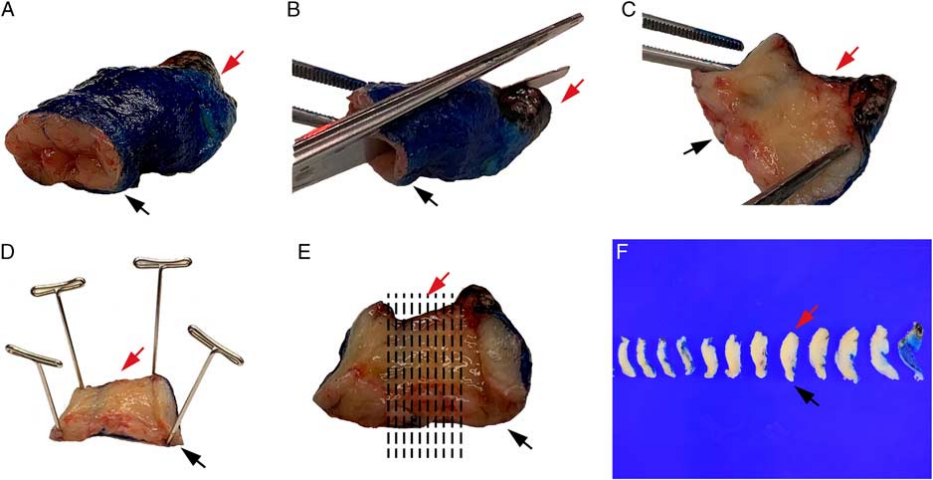
Fresh cone and loop electrosurgical excision procedure specimens can be opened and pinned out flat for formalin fixation before slicing. First the ectocervical (black arrow, A–F), deep connective tissue, and endocervical (red arrow, A–F) margins are inked. Use scissors to cut the cone open at 12 o’clock position (B) and lay flat (C), then pin before formalin fixation (D). Cut thin parallel slices perpendicular to the endocervical canal (E) to produce sections that each demonstrate the endocervical margin, the mucosa, the ectocervical margin and the deep connective tissue margin (F).

Ideally, a fresh intact LEEP/cone should be opened immediately and pinned out before formalin fixation as this strategy maximizes the opportunity for tissue sectioning to be performed in a way that preserves accurate tissue orientation, permits full visualization of the mucosa, and facilitates parallel 2 to 3 mm thick slices that do not need to be trimmed down before placement in tissue cassettes (Fig. 2). The disadvantage of this strategy is that is dependent on the availability of pathology laboratory staff to perform this step on receipt of the fresh specimen in order to prevent tissue autolysis. The procedure for opening a fresh intact LEEP/cone, as well as fixation and sectioning, is similar to that for a trachelectomy specimen, which is discussed below. If it is not feasible to open and pin a fresh intact specimen before formalin fixation, then the specimen should be placed in formalin upon receipt and processed as decribed below.

**FIG. 3:**
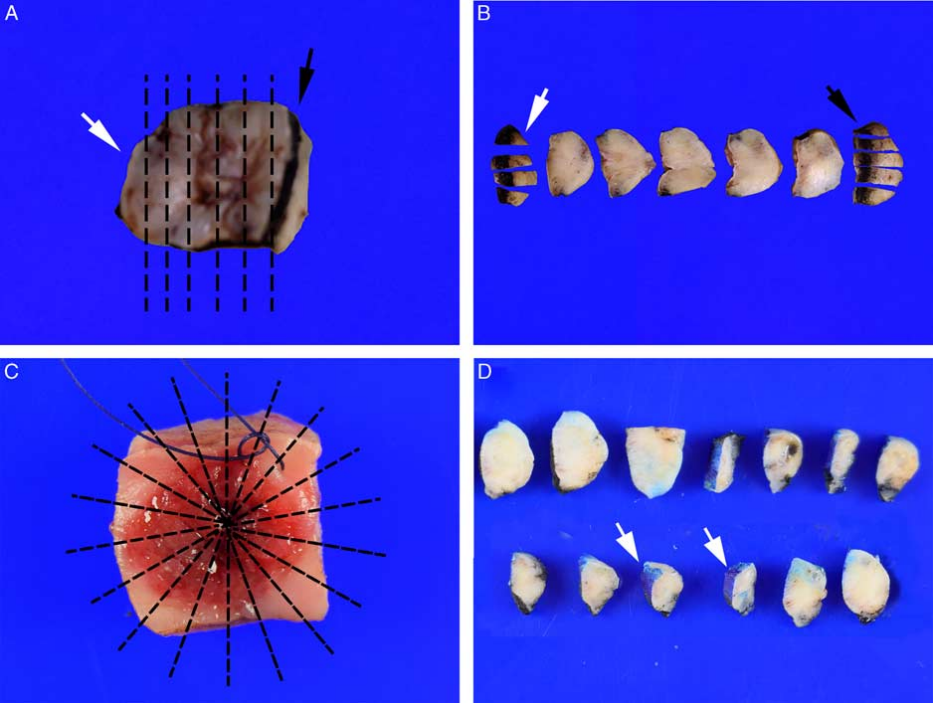
Formalin fixed cone and loop electrosurgical excision procedure specimens can be sliced either using a parallel slice strategy (A, B) or radial slice strategy (C, D). In the parallel slice strategy, the lateral slices (9 o’clock slice, white arrows; 3 o’clock slice, black arrows, A, B) can be cut further and placed on edge to produce perpendicular margins. This slicing strategy produces uniformly thin sections that do not need to be trimmed down to lay flat in the tissue cassettes. Alternatively, the radial slice strategy (C, D) produces wedge-shaped sections (white arrows, D) that need to be trimmed so that the sections can be placed flat into tissue cassettes.

If the LEEP/cone is left intact during formalin-fixation, then there are 2 options for processing the specimen: radial slicing or parallel slicing (Fig. 3). The radial strategy is best suited for specimens in which the endocervical canal is clearly visible, whereas specimens in which the endocervical canal is less obvious may be better managed by parallel slicing. For the radial strategy, the goal is that each slice should have mucosa from the ectocervical margin to the endocervical margin along one edge of the section and the deep connective tissue margin at the opposite edge. It is unavoidable that the radial strategy produces tissue sections that are thicker toward the deep connective tissue part of the slice, creating a wedge-like shape that will not lay down flat in the tissue cassette. Therefore, the excess tissue at the thicker end of the section should be trimmed to produce a flat section. These trimmed pieces will not contain any mucosa but should still be examined microscopically. For the parallel slicing strategy, the specimen is serially cut in 2 to 3 mm thick slices parallel to the endocervical canal. This produces uniform thin slices that do not need to be trimmed down further. To fully evaluate the mucosal and connective tissue margins of the first and the last slices, these can be further cut perpendicularly and embedded on their sides.

All tissue should be submitted for microscopic examination, including the excess trimmed tissue. The sections should be placed in consecutive cassettes and the cassette code should document the anatomic orientation of the slices in order to facilitate tumor size measurements in the event that tumor is present in >1 slice. Only 1 to 2 sections should be submitted in each cassette.

On occasion, a LEEP specimen may be accompanied by an additional, usually smaller, cylinder of endocervix, the so-called top hat specimen, when the clinician determines that additional tissue is necessary to assure a negative margin. The length, diameter and wall thickness should be documented. The true endocervical margin and deep connective tissue margin should be inked. The specimen can be sliced either using a radial approach or parallel approach, as described for the main LEEP specimen. The entire tissue should be submitted for microscopic examination.

Regarding the number of initial H&E-stained sections per block to examine, the recommendations stated for fragmented LEEP specimens apply to intact LEEP and cone specimens.

#### Recommendations

- Fresh intact LEEP/cone specimens can either be opened and pinned before fixation or placed intact in formalin, depending on local practice resources.
- Specimens opened and pinned before fixation should be thinly sliced parallel to the endocervical canal.
- Specimens fixed intact can be sliced using a radial or a parallel slicing strategy.
- Specimens should be entirely submitted for microscopic examination, including any excess trimmed pieces.
- If an additional so-called top hat specimen is submitted, it should be inked, sliced using the same strategy for the main LEEP specimen, and entirely submitted.
- A single H&E-stained section per block is sufficient for initial microscopic examination, with consideration of deeper sections when there is missing mucosa, absence of squamous intraepithelial lesion/endocervical adenocarcinoma, suspicion for stromal invasion, or findings that are discordant with the clinical, colposcopic, and/or cytologic findings. Alternatively it may be more efficient to routinely examine 2 or more sections per block, depending on the local practice.

## TRACHELECTOMY SPECIMENS

Trachelectomy is a fertility sparing approach for selected stage I cervical cancers (stage IA1 with lymphovascular space invasion, IA2, or IB with clinically estimated cervical length of 2 cm or less and no radiologic evidence of tumor involvement of the upper endocervix or any extrauterine site) 6–9. Endocervical adenocarcinoma is slightly more prevalent than squamous cell carcinoma among trachelectomy patients in some studies 10,11. Experience remains limited in patients with tumors larger than 2 cm and/or postneoadjuvant therapy 12–14. Radical trachelectomy consists of the cervix (ectocervix, transformation zone, and endocervical canal), upper vagina (cuff of 1–2 cm), and lower parametria. Simple trachelectomy does not include the parametria. Trachelectomy specimens are usually received intact and fresh from the operating room. They are typically submitted for intraoperative consultation, which requires immediate orientation, inking and measuring. After intraoperative sampling and processing, the specimen can be pinned for formalin fixation.

### Specimen Orientation and Inking

**FIG. 4:**
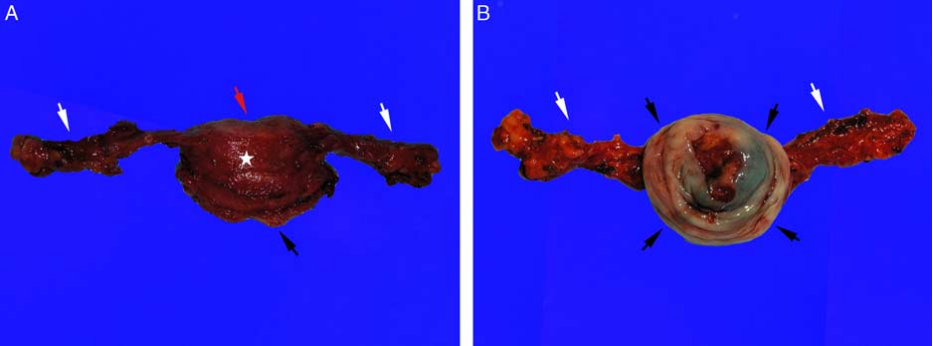
Radical trachelectomy specimen viewed from anterior surface (A) and from en face of the cervix (B). Bilateral parametrial tissues (white arrows) should be inked. The vaginal cuff (black arrows) may retract around the cervix and should be stretched out before measuring its length and before pinning for formalin fixation. The endocervical margin (red arrows) should be inked as well as the connective tissue at the outer surface of the anterior wall (white star) of the cervix and the posterior wall.

Identification of the endocervical and vaginal margins of the specimen is usually straightforward based on the appearance of the vaginal cuff (Fig. 4). The anatomic orientation of the specimen in the axial plane allows the parametrial tissue to be oriented as right or left. This is dependent on the surgeon to place an orientation suture (eg, designating 12 o’clock position using a clock-face system; designating the anterior lip of the cervix; or designating the right vs. left parametrial tissue).

The nonperitonealized connective tissue at the outer surface of the anterior and posterior cervical walls is not a true surgical margin though this is often referred to as a radial or paracervical margin 15. Nevertheless, as the status of this surface (involved by tumor vs. not involved by tumor) may be of relevance to the surgeon and/or radiation oncologist 16 it is advised to ink these surfaces and document presence or absence of tumor.

The margins to ink are the endocervical, vaginal, and parametrial margins, as well as the nonperitonealized connective tissue at the outer surface of the anterior and posterior cervical walls.

#### Recommendations

- Orient the laterality of the parametria and the anterior/posterior lip of the cervix based on the orientation provided by the surgeon.
- Ink the endocervical, vaginal, and parametrial margins, as well as the nonperitonealized connective tissue at the outer surface of the anterior and posterior cervical walls.

### Specimen Measurements

It is recommended to document the measurements of the cervix, vaginal cuff, and parametria. As the vaginal cuff may retract around the cervix, it should be stretched out before taking measurements.

#### Recommendations

- Measure the cervix length (parallel to the endocervical canal), diameter and wall thickness.
- Measure the parametrial tissue length (from superior to inferior) and lateral dimension (from uterine wall to outer edge).
- Measure the vaginal cuff minimal and maximal length after stretching it out if it is retracted.

### Tumor Location

Documenting the anatomic location of grossly visible tumor assists in the pathologic correlation with radiologic and intraoperative findings, particularly if there is clinical concern for margin involvement by tumor. We recommend using positions on a clock face and then correlate with the anatomic terminology used by the surgeon to designate the tumor location. If grossly visible tumor invades the cervical wall, the greatest depth of invasion should be documented as well as the total thickness of the cervical wall at that point.

#### Recommendations

- Document the anatomic location of tumor in the cervix using positions on a clock face and then correlate with the anatomic terminology used by the surgeon.
- Document the distance of tumor to the endocervical margin, vaginal margin, parametrial margin, and nonperitonealized connective tissue at the outer surface of the anterior and posterior cervix walls.

### Macroscopic Tumor Dimensions

A unified approach to determine tumor size measurements in cervical cancer is critical for several reasons: There is significant variation in the method used by gynecologic oncologists, radiologists, and pathologists to estimate tumor size, and currently there is no single recommendation for standard practice. Importantly, the current FIGO staging system for cervical cancer recognizes pathologic variables to influence stage. Moreover, tumor size measurements taken by the pathologist supercede those obtained clinically or radiologically 17. Size measurements should be obtained in the fresh specimen. The distance to the margins should also be measured at this time. Measuring the lesion and its distance to margins after fixation and handling (opening, pinning) is discouraged for 2 reasons. First, it will likely lead to overestimation of tumor size, as the specimen will be stretched out. Second, there is conflicting evidence on the effect of fixation on specimen and tumor size, with several studies reporting shrinkage after fixation 18,19 while others report no significant differences 20,21.

**FIG. 5:**
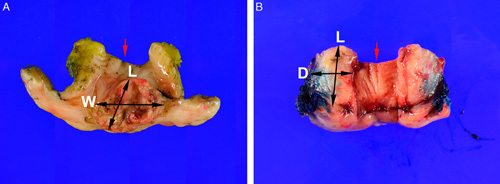
Tumor length (L) is defined as the dimension parallel to the endocervical canal while tumor width (W) is the plane perpendicular to the endocervical canal. The tumor depth cannot be appreciated in the specimen in image A (A) until the specimen is sectioned further. The tumor in (B), a different specimen, diffusely replaces the wall of the cervix circumferentially without exhibiting an exophytic component. Tumor depth (D) is defined as the dimension from the endocervical mucosa through the cervical wall towards the outer connective tissue surface. Image A is a trachelectomy (red arrow is endocervical margin). (B) A radical hysterectomy (red arrow is site of amputation from uterine corpus).

The 3 macroscopic dimensions of a cervical tumor are its length (parallel to the endocervical canal), width (in the plane perpendicular to the endocervical canal), and thickness (from tumor surface to the tumor deepest invasion point) (Fig. 5). The macroscopic depth of invasion is defined as the distance from the endocervical mucosa to the tumor deepest point within the cervical wall. Depending on the relative amount of exophytic growth versus growth into the cervical wall, tumor thickness and tumor depth of invasion may be different.

#### Recommendations

- Document the tumor length (parallel to the endocervical canal), tumor width (in the plane perpendicular to the endocervical canal), tumor thickness and depth of tumor invasion.

### Specimen Processing and Tissue Sampling

Open the trachelectomy specimen by making one cut through the wall at one anatomic location (eg, at the 12 o’clock position), parallel to the endocervical canal. If possible, the cut should be perfomed in an area free of gross tumor, though this may not always be possible. This will convert the intact cylindrical shape of the specimen into a rectangle that can be pinned out flat for formalin fixation and will permit the tissue to be sliced in a way that maximizes the preservation of the orientation of all margins for microscopic examination. This will also permit the vaginal cuff, which normally retracts around the ectocervix, to be stretched out before fixation so that the true distance between this margin and the tumor can be evaluated; otherwise, tissue retraction may lead to a significant underestimate of the length of the vaginal cuff that the surgeon resected. Removing the parametrial tissues before opening and pinning the trachelectomy is advised. Pins should be placed in a way that does not disrupt the mucosal margins or the tumor itself. Adequate formalin-fixation will facilitate optimal tissue sectioning. Overnight fixation may be needed.

After fixation, the trachelectomy specimen should be serially sliced at 2 to 3 mm intervals parallel to the endocervical canal. Each slice should have mucosa along one edge (from the vaginal cuff margin to the endocervical mucosal margin) and the radial paracervical connective margin along the other edge. If the slices are too large to fit in a cassette, they can be divided into 2 or 3 sections and placed in consecutive cassettes. Large format (macro) blocks, if available, may be of value in such cases.

If tumor is grossly visible, sampling should be focused to document the deepest point of invasion as well as the closest distance to all margins. Aside from these key anatomic landmarks, there is no evidence to guide whether the entire tumor or representative sections should be submitted for microscopic examination. From the practical standpoint of tumor stage assignment, it is recommended to entirely submit tumors that are 2 cm or less since microscopic examination may affect the final tumor dimensions, particularly if macroscopically evident wound-healing changes are present, which may lead to over or under-estimation of true tumor size. Tumors larger than 2 cm can be sampled with representative sections. If tumor is not grossly visible then the entire specimen should be submitted for microscopic examination.

The vaginal margin should be examined by a perpendicular section at the site of the closest approach of the tumor. In such a case, there is variability across practices as to whether the remainder of the vaginal margin should be examined entirely en face or whether an additional representative perpendicular section away from the tumor is sufficient. If there is no macroscopic tumor, there is also variability among practices as to whether the entire vaginal margin should be examined en face or whether representative perpendicular sections are sufficient.

The parametria should be thinly sliced and entirely submitted, preserving the right and left side orientation. If lymph nodes are present within parametria, their number, size, and appearance should be documented 22.

A single H&E-stained section per block is sufficient for the initial microscopic examination.

#### Recommendations

- Remove parametria and place in cassettes before opening specimen.
- Open the specimen and obtain measurements (anatomic structures and any lesion) immediately upon receipt.
- After intraoperative consultation (if performed), pin the specimen for overnight formalin fixation, taking care to stretch out the vaginal cuff to its full length and pin.
- Tumors 2 cm or less should be entirely submitted while tumors larger than 2 cm can be processed using representative sections.
- Tissue sections should demonstrate the deepest tumor invasion and the closest approach of the tumor to the vaginal, radial, and parametrial margins.
- If there is no grossly visible lesion, the entire cervix should be submitted.
- Perpendicular sections of the vaginal margin closest to the tumor should be examined. Whether the remainder of the vaginal margin should be examined entirely en face or by representative perpendicular sections is left to local practice standards. Similarly, if there is no macroscopic tumor, the decision to examine the entire vaginal margin en face or by representative perpendicular sections is left to local practice standards.
- The parametria should be entirely submitted.
- A single H&E-stained section per block is sufficient for initial microscopic examination.

### Intraoperative Evaluation

#### Clinical Indications

In the first descriptions of the trachelectomy procedure, an endocervical margin at least 8 to 10 mm away from the tumor was considered optimal. Conversely, a positive margin or a negative margin <5 mm from the tumor would be considered insufficient, prompting additional surgery 6,7. For this reason, intraoperative evaluation of the proximal (endocervical) margin is routinely performed in this setting. Margin status is critical in the management of these patients: (1) recurrence rates are influenced by margin status, and (2) while the pregnancy success rate is high in these patients, the pregnancy is at risk of complications such as prematurity and first-trimester miscarriage 23. For these reasons, preservation of as much of the proximal canal as possible is imperative.

#### Intraoperative Impact of Findings

The status of the endocervical (proximal) resection margin determines the need for additional excision 24,25. If the proximal margin is positive for invasive carcinoma, radical hysterectomy would be considered. Alternatively, if feasible, an additional portion of the upper endocervix will be removed. If the proximal margin is negative, but the distance of the margin to invasive tumor is <5 mm, an additional portion of the upper endocervix will be removed to guarantee an appropriate width of margin excision.

The status of the deep (paracervical) and vaginal mucosa margins is usually not required intraoperatively.

#### Specimen Processing

Four different strategies for intraoperative tissue handling and sectioning have been proposed (Table 1) 24,25,27,28, each with excellent sensitivity and negative predictive value. In the absence of any evidence directly comparing these strategies, it is recommended that the protocol of choice be defined at the local practice level, in conjunction with the surgeon to understand their specific intraoperative needs from the pathologic evaluation of the trachelectomy intraoperatively.

**TABLE 1:** Comparison of published protocols for intraoperative examination of trachelectomy specimens

	Tanguay et al. 25; Chênevert et al. 26	Park et al. 27	Ismiil et al. 28	Zhang et al. 29
Number of patients	53	19	132	53
Adenocarcinoma	Not specified	19	63	11
Squamous cell carcinoma		0	59	40
Others (adenosquamous, etc.)		0	7	2
Concordance between FS and permanent sections	91%	95%	99%	100%
False positive rate	0%	5%	1%	0%
False negative rate	9%	0%	0%	0%
Brief description of the protocol	No frozen section on normal-looking cervix If lesion present, one longitudinal section including tumor and margin	En face shaved proximal margin, 2 mm thick If positive for carcinoma; no further work-up required If negative: 1. If tumor present, report gross distance 2. If tumor uncertain, perpendicular section 3. If no tumor present, second transverse section at 10 mm from the proximal margin taken	Proximal 1 cm cervical segment is cut-off and serially sectioned clockwise into 10–12 perpendicular sections Each section is processed for frozen section If sections harbor carcinoma, the distance to the proximal margin is measured	One transverse section 2 mm thick, 8 mm distal to the proximal margin If section is negative, no further processing If section positive, an additional segment of the endocervix is removed and a new transverse section 8 mm from the revised proximal margin is submitted for frozen section
Advantages	Provides exact distance to the margin Few frozen sections (1–2)	Provides exact distance to the margin Few frozen sections (1–4)	Provides exact distance to the margin Excellent PPV and NPV Validated in >100 cases	Few frozen sections (1–2) Excellent PPV and NPV
Disadvantages	9% false positive rate Validated in <100 cases	5% false positive rate Validated in <100 cases	Time and labor intensive (10–12 frozen sections)	Does not provide exact distance to the margin Validated in <100 cases

NPV indicates negative predictive value; PPV, positive predictive value.

#### Reporting Terminology

The margin status should be reported as either positive or negative for invasive carcinoma and for in-situ carcinoma. If the margin is negative, the distance of the closest approach of tumor should be reported. In sections taken perpendicularly, a positive margin is defined as invasive carcinoma in direct contact with the inked surface of the margin; all other instances are defined as a negative margin. In *en face* margin sections, a positive margin is defined as invasive carcinoma present in the section, and a negative margin as absence of invasive carcinoma in the section.

#### Challenges With Interpretation, Reporting and Diagnostic Pitfalls

*Margin Artifacts (Folding, Tissue Gaps, Irregularities).* Intraoperative frozen section examination of the endocervical margin requires good visualization of the entire wall thickness and the inked margin edge (if section is perpendicular). If the initial section is significantly folded or fragmented, obtaining a new level is highly advisable. Identification of tissue gaps or incision marks before inking is very important, as they may distort the endocervical margin. Ink must be applied carefully, avoiding surfaces that do not represent resection margins. Examination of the specimen with the surgical team may be required.

*Benign Mimickers.* Mimickers of endocervical adenocarcinoma include tubo-endometrioid metaplasia and endometriosis. In addition, the proximal margin of the trachelectomy specimen may sometimes be at the lower uterine segment or even endometrium functionalis. These scenarios feature mucin-depleted glands and variable degrees of nuclear pseudostratification as well as proliferation, thus highly resembling human papillomavirus (HPV)-related adenocarcinoma. In addition, tubo-endometrioid metaplasia can feature reactive stroma, further raising concern for malignancy. Attention to the nuclear characteristics is important. In the presence of bland nuclear features), presence of cilia as well as terminal bars, and/or periglandular endometrial-type stroma and/or hemorrhage, a benign diagnosis should be entertained 30,31.

A scoring system to distinguish endocervical adenocarcinoma in situ from benign/reactive conditions has been published and subsequently applied to intraoperative evaluation of trachelectomy specimens 27,32. When narrowed to 2 final diagnoses (benign/reactive vs. adenocarcinoma), this system had 94% concordance with the index diagnoses and, when used intraoperatively on trachelectomy specimens, improved the positive predictive value of frozen section and the concordance rate between the intraoperative and final diagnoses.

#### Recommendations

- The protocol for intraoperative evaluation of trachelectomy specimen should be decided at the local practice level using 1 of the 4 published protocols 24,25,27,28 (Table 1).
- The presence or absence of invasive cancer and of in situ carcinoma at the proximal margin (defined as ink on tumor) should be reported for the intraoperative evaluation. If the margin is negative, then the distance between tumor and margin should be reported.

## HYSTERECTOMY SPECIMENS

There are 3 types of hysterectomy performed for cervical cancer: radical, modified radical, and simple (extrafascial) 9. Radical hysterectomy is the default approach for FIGO stage IA2, IB1, IB2 and select IB3-IIA1 cervical cancers in women who do not desire fertility preservation. Radical hysterectomy includes the uterine corpus, uterine cervix, the upper 1 to 2 cm of the vagina and bilateral parametrial connective tissue. Parametrectomy is a key surgical goal of radical hysterectomy given the overall risk for microscopic parametrial involvement by cervical cancer, which carries adverse prognostic significance 33–35. Modified radical hysterectomy may be considered in some women with stage IA1 with lymphovascular space invasion or stage IA2 cervical cancer. The modified procedure is less extensive. As the risk for parametrial involvement is significantly lower for early stage cervical cancer, less radical surgery has been offered to selected patients (stage IA1 without lymphovascular invasion) using simple hysterectomy without parametrectomy or upper vaginectomy 36,37. In such specimens, there may be some connective tissue attached to the cervix, which should be examined microscopically, but not reported as formal parametrial tissue 38.

The ovaries and/or fallopian tubes may be included with hysterectomy in certain clinical scenarios. Involvement of the fallopian tubes is rare but up to a quarter of patients with endocervical adenocarcinoma may have ovarian metastasis, particularly in the setting of involvement of the uterine corpus, parametrium, and/or lymphovascular spaces in the cervix 39–41.

### Specimen Orientation, Margins, and Inking

**FIG. 6:**
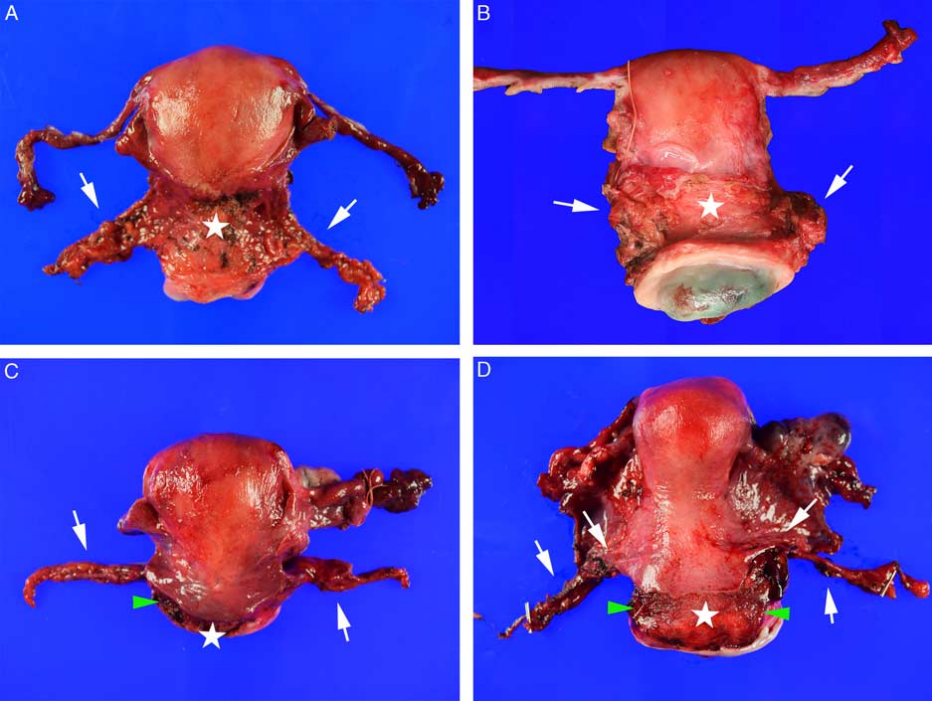
The parametrial tissue (white arrows, A–D) at the lateral sides of a radical hysterectomy may vary in size and shape. It may retract (B) and should be stretched out to ink, measure and dissect. The nonperitonealized connective tissue (white stars, A–D) at the anterior and posterior surfaces of the cervical wall should be inked to assess for tumor involvement. In some cases (C, D) a small rim of connective tissue that is not part of the parametrial tissue may also be present at the lateral surfaces of the cervical wall (green arrowheads, C, D) and should be inked. The vaginal cuff in all 4 images (A–D) has retracted around the cervix and should be stretched out before measuring and pinned out for formalin fixation (refer to Fig. 7).

Two anatomic landmarks permit orientation of a hysterectomy specimen: (1) the peritoneal reflection is shorter on the anterior surface of the uterus because of the normal position of the urinary bladder anterior to the uterus, whereas the peritoneal reflection extends further down the posterior aspect of the uterus. (2) The round ligaments are located anterior to the fallopian tubes (Fig. 6).

The nonperitonealized connective tissue at the outer surface of the anterior and posterior cervical walls is not a true surgical margin though this is often referred to as a radial or paracervical margin (Fig. 6) 15. Nevertheless, the status of whether tumor extends to this surface may be of relevance to the surgeon and/or radiation oncologist 16 and so it is advised to ink these surfaces and document presence or absence of tumor involvement.

The margins to ink are the vaginal and parametrial margins, as well as the nonperitonealized connective tissue at the outer surface of the anterior and posterior cervical walls.

#### Recommendations

- Ink the vaginal and parametrial margins, as well as the nonperitonealized connective tissue at the outer surface of the anterior and posterior cervical walls.

### Specimen Measurements

**FIG. 7:**
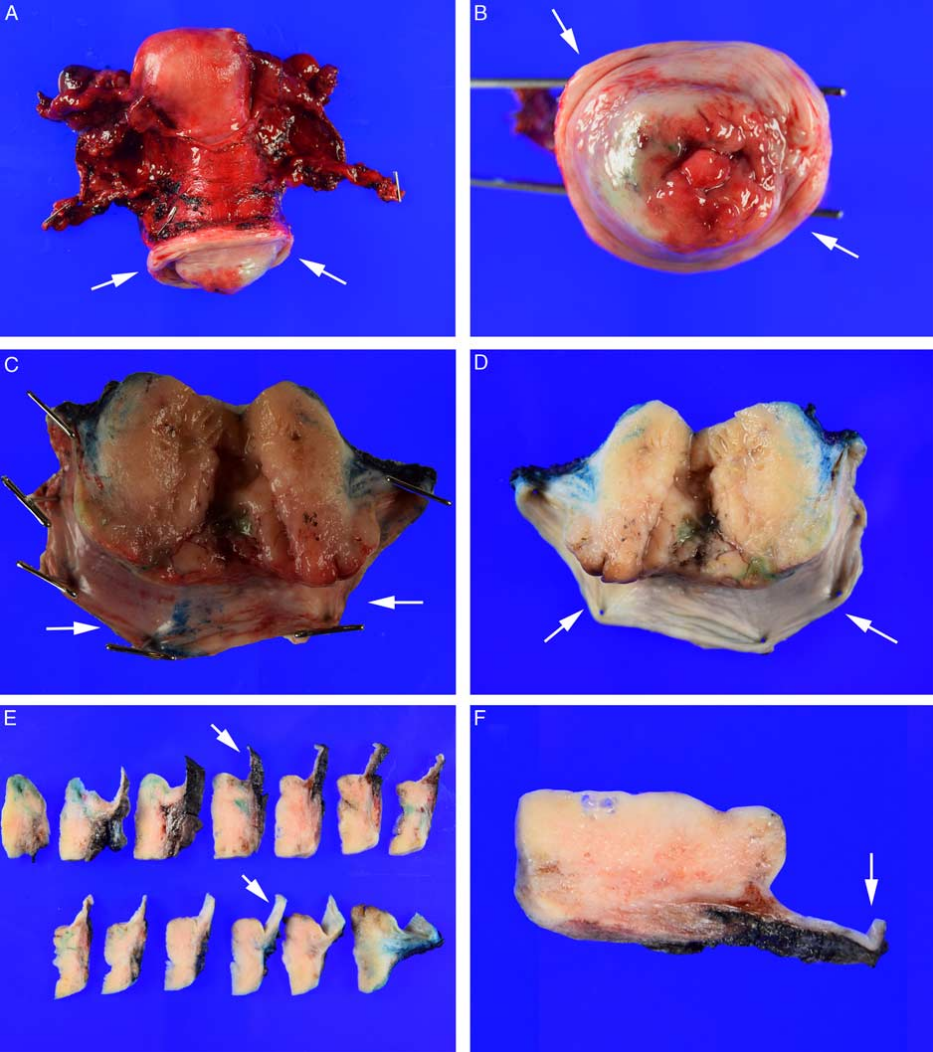
The vaginal cuff (arrows, A–F) on a radical hysterectomy may retract around the cervix (A, B), which may cause under-reporting of the true measurement of the cuff length and of the distance from tumor to the cuff margin. Therefore, the vaginal cuff should be stretched out to its full length and pinned before formalin fixation (C). This preserves the true dimensions of the vaginal cuff (D) and permits slicing the formalin fixed cervix (E) while preserving the relationship of the vaginal cuff margin to tumor and allowing for accurate assessment of the margin status (F).

It is recommended to document the measurements of the cervix, vaginal cuff, parametria, uterine corpus, and, if present, the ovaries and tubes. As the vaginal cuff may retract around the cervix, it should be stretched out before taking measurements (Fig. 7).

Although there is no clinically relevant role for recording the hysterectomy specimen weight, it is routinely recorded in pathology practices in the United States since there are different billing codes for specimens that weigh up 250 g versus those above it 42. Furthermore, the certification requirements of the American Board of Obstetrics and Gynecology mandate candidates to document specimen weights during their training experience in performing hysterectomies. For these reasons, pathology practices in the United States typically document the weight of hysterectomies, regardless of the clinical setting. As such conditions may not necessarily apply in other countries, documentation of specimen weight is regarded as an optional recommendation defined by the local practice conditions.

#### Recommendations

- Weight the uterus.
- Measure the cervix length (parallel to the endocervical canal), diameter, and wall thickness.
- Measure the parametrial tissue length (from superior to inferior) and lateral dimension (from uterine wall to outer edge).
- Measure the vaginal cuff minimal and maximal length after stretching it out if it is retracted.
- Measure the uterine corpus from superior to inferior, side to side and anterior to posterior dimensions.
- If present, the size of the ovaries and fallopian tubes should be recorded.

### Strategy for Opening the Hysterectomy

If there is no suspicion that the tumor is extending into the parametria, then they can be removed at the interface with the uterine wall before opening the uterus. The parametria are then sliced at 2 to 3 mm intervals and placed in casettes immediately to ensure that these tissues are entirely submitted and that their right/left orientation is preserved. If there is suspicion of tumor extension into the parametria then it is recommended to leave the parametria attached in order to permit slicing of the cervix and parametria in continuity to demonstrate direct tumor extention.

Hysterectomy specimens should be opened immediately upon receipt in the pathology laboratory and prepared for formalin-fixation in order to mitigate tissue autolysis, which may impair microscopic examination and/or immunohistochemical and molecular testing 43–46. Even if the hysterectomy is received by the laboratory already in formalin, the endocervical and endometrial lining, as well as any tumor, are the least likely parts of the specimen to be exposed to the formalin if the uterus was not opened. Thus, such specimens should also be opened immediately upon receipt in the pathology laboratory.

**FIG. 8:**
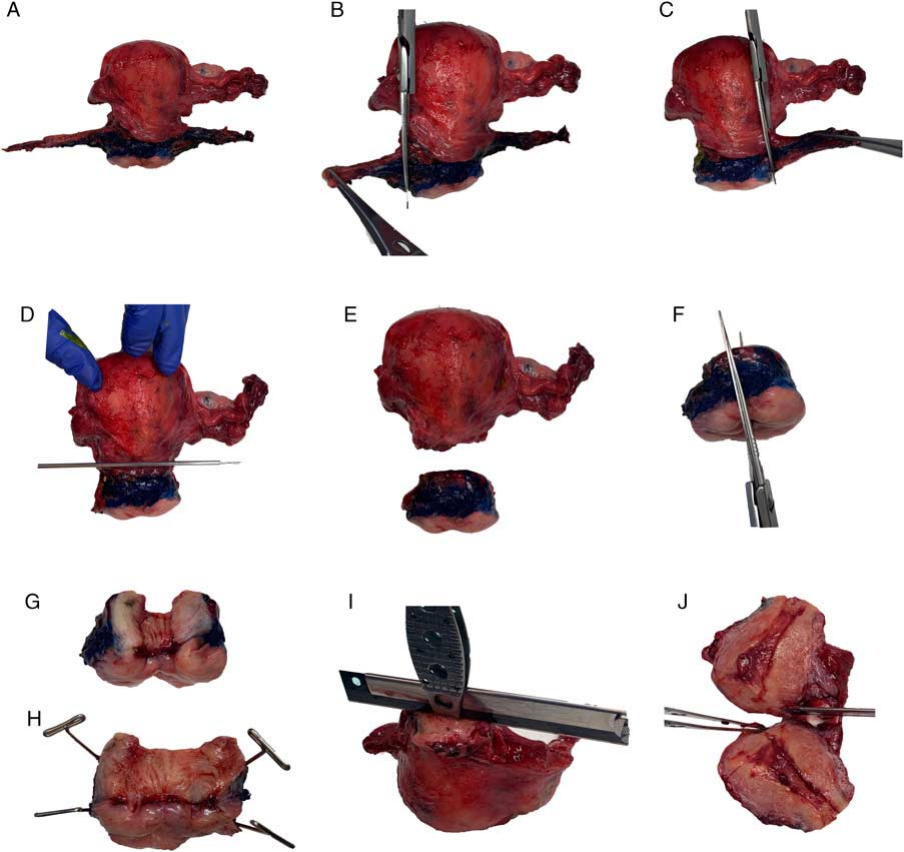
Dissection of a fresh radical hysterectomy using the strategy of cervical amputation begins with inking of the parametrium, paracervical connective tissue and vaginal cuff margins (A). If there is no suspicion for tumor extension into the parametrium, then the parametrium from each side is removed with scissors (B, C) and the cervix is amputated (D) from the uterine corpus (E). The amputated cervix is opened with scissors by cutting through the wall at the 12 o’clock position (F). The resulting opened cervix (G) is pinned out flat for formalin fixation (H). The strategy for slicing and sampling the opened cervix after fixation is the same as for an intact cone/loop electrosurgical excision procedure LEEP or trachelectomy specimen (Fig. 2). The uterine corpus is opened either by using scissors along the lateral borders (not shown) or by placing forceps into the endometrial cavity and guiding a knife between the forceps arms (I) to bivalve the specimen into anterior and posterior halves (J).

If the specimen is received fresh, 2 options exist to open the uterus. The first consists of amputation of the uterine cervix from the corpus and process the cervix using the same strategy as for an intact cone or trachelectomy (Fig. 8). The uterine body is then opened along the lateral walls using the conventional bivalve approach, resulting in an anterior and a posterior half. The advantage of this strategy is that it permits well-oriented, uniformly thin slices of the cervix to be cut, which facilitates optimal microscopic evaluation for invasion and margin assessment. The disadvantage is that this requires laboratory staffing to be available to perform this processing immediately upon receipt of the fresh specimen. The second option is to use the conventional bivalve approach for opening a uterus along the lateral wall resulting in an anterior half and a posterior half 42, and can also be used if the hysterectomy is received in formalin. The disadvantage is that the cervix will have to be dissected using a radial slice strategy, similar to that for a formalin-fixed intact cone, which produces slices of uneven thickness that have to be trimmed down to fit in the cassette properly. The decision is left to each local practice as to which of these 2 strategies to use.

The uterine corpus should be serially thinly sliced (3–5 mm) parallel to the axial plane, from the endometrial surface through the wall to the serosa, as is conventionally done for other hysterectomy indications 42. Careful evaluation for secondary involvement of the corpus is merited given its association with ovarian and/or lymph node metastasis.

Once the uterus is opened using either strategy above, immediate formalin-fixation is advised before conducting any further tissue sampling. In addition to preventing the consequences of tissue autolysis, formalin-fixation facilitates cutting thin, well-oriented tissue slices of the cervix and tumor, which in turn facilitates accurate microscopic assessment of tumor dimensions and distances to margins. Fixation times vary depending on the size of the specimen but a practical approach is to permit the specimen to fix overnight and complete the dissection and tissue sampling the next day.

The vaginal cuff, which may retract around the cervix, should be stretched out before pinning.

#### Recommendations

- If there is no suspicion that the tumor is extending into the parametria, then they can be removed at the interface with the uterine wall, sliced at 2 to 3 mm intervals and placed in tissue cassettes before opening the uterus. Otherwise the parametria should be left attached and sliced in continuity with the cervix.
- Open the uterus immediately upon receipt in the lab in order to begin formalin fixation.
- Fresh hysterectomy specimens can be opened either by amputating the cervix and processing it like a trachelectomy or by the conventional bivalve strategy for opening a uterus.
- Slice the uterine corpus in parallel thin slices before formalin fixation.
- Stretch out the vaginal cuff to its full length and pin in position before formalin fixation.
- Overnight formalin fixation is advised before further tissue sampling.

### Tumor Location

Documenting the anatomic location of grossly visible tumor assists in the pathologic correlation with radiologic and intraoperative findings, particularly if there is clinical concern for margin involvement by tumor. We recommend using positions on a clock face and then correlating with the anatomic terminology used by the surgeon. If grossly visible tumor invades the cervical wall, the greatest depth of invasion should be documented as well as the total thickness of the cervical wall at that point. This permits correlation with microscopic measurements in order to report tumor involvement of the inner, middle, and/or outer third of the cervical wall. This 3-tier system of assessing cervical wall involvement is part of the Sedlis criteria, along with tumor size and lymphovascular space invasion status, used to determine eligibility for external pelvic radiation in cervical cancer patients whose radical hysterectomy shows node-negative, margin-negative, and parametria-negative disease 47–49.

If parametrectomy is performed, it should be documented whether tumor is confined to the cervix or involves the parametrium. Tumor involvement of the uterine corpus is important to document as it is associated with increased risk of ovarian and para-aortic lymph node metastasis 39–41.

The distance of tumor to the vaginal cuff margin, parametrial margin, and nonperitonealized connective tissue at the outer surface of the anterior and posterior cervix walls should be recorded.

#### Recommendations

- Document tumor involvement in relation to the endocervix, ectocervix, parametria, and uterine corpus.
- Document the anatomic location of tumor in the cervix using positions on a clock or designating anterior versus posterior lip of the cervix.
- Document distance of tumor to the vaginal margin, parametrial margin, and nonperitonealized connective tissue at the outer surface of the anterior and posterior cervix walls.

### Macroscopic Tumor Dimensions

The recommended measurements of the gross specimen and tumor (if grossly visible) are listed in Table 2.

**TABLE 2:** Recommended macroscopic measurements of radical hysterectomy for cervical cancer

Specimen measurements
Overall specimen
Weight of uterus
Dimensions (fundus to cervix; anterior to posterior; cornu to cornu)
Cervix
Diameter of ectocervix
Thickness of wall
Vaginal cuff
Minimum length
Maximum length
Parametrial dimensions (record separately for each side)
Lateral dimension (from uterine wall to outer edge)
Length (dimension along axis from fundus to cervix)
Corpus
Thickness of endometrium
Thickness of myometrium
Ovaries and fallopian tubes (if present)
Dimensions of short axis and long axis of ovary
Length and diameter of fallopian tube
Tumor location
Anatomic position
Involvement of endocervix, ectocervix, uterine corpus, and/or parametrium
Location in cervix (using positions on a clock face)
Distance to margins
Distance to closest vaginal cuff margin
Distance to closest parametrial margin
Distance to nonperitonealized connective tissue of outer surface of anterior and posterior cervical walls
Tumor dimensions
Length (parallel to endocervical canal)
Width (perpendicular to endocervical canal)
Thickness (from tumor surface to tumor deepest edge)
Depth of invasion
Thickness of cervical wall at point of deepest invasion by tumor

As stated in the trachelectomy section, a standardized approach to document tumor size is critical. Tumor dimensions and distance to the margins should be obtained in the fresh specimen, before pinning, stretching and fixation. The 3 macroscopic dimensions of a tumor are its length (parallel to the endocervical canal), width (in the plane perpendicular to the endocervical canal), and thickness (from tumor surface to the tumor deepest invasion point). The macroscopic depth of invasion is defined as the distance from the endocervical mucosa to the tumor deepest point within the cervical wall. Depending on the relative amount of exophytic growth versus growth into the cervical wall, tumor thickness and tumor depth of invasion may be different.

Of note, documentation of macroscopic tumor dimensions in the report is not required as mandatory by the International Collaboration on Cancer Reporting (ICCR) or from the College of American Pathologist’s (CAP) Cancer Reporting Protocol 15,38. However, for practical purposes it is recommended to record macroscopic length, width, thickness, and depth of invasion in the gross description since these data are eventually needed to determine the final dimensions after review of the microscopic findings. For the purposes of reporting and assigning pathologic tumor stage, only a single final value for each dimension should be given. This final size should be based on (a) correlating the macroscopic and the microscopic measurements in the hysterectomy specimen; (b) integrating the tumor dimensions in the preceding LEEP or cone specimen, which may be larger than those in the hysterectomy specimen; and (c) integrating pretherapy clinical and radiologic tumor dimensions if chemotherapy and/or radiation was administered before hysterectomy as these may also be larger than those in the hysterectomy specimen. Details on determining the final tumor dimension are discussed in the separate review in this issue on tumor staging recommendations.

#### Recommendations

- Document the tumor length (parallel to the endocervical canal), tumor width (in the plance perpendicular to the endocervical canal), tumor thickness and depth of tumor invasion.

### Specimen Processing and Tissue Sampling

If the cervix was amputated and processed like an intact cone or trachelectomy, then it should be serially sliced at 2 to 3 mm intervals parallel to the endocervical canal. Each slice should have mucosa along one edge (from the vaginal cuff margin to the endocervical mucosal margin) and the paracervical connective tissue surface along the other edge. If the slices are too large to fit in a cassette, they can be divided into 2 or 3 sections and placed in consecutive cassettes. Large format (macro) blocks, if available, may be of value in such cases. Alternatively, if the hysterectomy was opened using the bivalve approach, then the radial slice approach should be used, similar to that used for a formalin-fixed cone/LEEP specimen. Serial 2 to 3 mm slices are made parallel to the endocervical canal. Because of the half-cylindrical shape of the fixed cervix, this will create wedge shaped slices that are thicker at the outer cervical wall. These wedge shaped slices will have to be trimmed so they lay flat in the tissue cassette.

Sampling of any grossly visible tumor follows recommendations stated in the trachelectomy section. In short, it is recommended to entirely submit tumors that are 2 cm or less, and representatively sample tumors larger than 2 cm. If tumor is not grossly visible then the entire cervix should be submitted for microscopic examination.

Sampling of the vaginal margin is as recommended for trachelectomy specimens.

The uterine corpus and lower uterine segment should be examined for tumor involvement. In addition to sampling any gross abnormalities, representative sections of the full thickness (ie from endometrium to serosa) of the lower uterine segment (in respect to any visible lesion) and the anterior and posterior walls of the corpus is recommended.

If salpingectomy and/or oophorectomy were performed, these organs should be sliced using the sectioning and extensive examination of the fimbriae (SEE-Fim) protocol to evaluate for metastasis, which may occur, albeit uncommonly 39–41,50. There is no clear evidence to guide whether grossly normal appearing ovaries and fallopian tubes should be microscopically examined in their entirety in this setting. At a minimum it is recommended that the entire fimbriae of each fallopian tube be examined microscopically along with representative sections of the ampullary portion of the fallopian tubes, representative sections of the ovaries, and sampling of any abnormalities.

#### Recommendations

- If the cervix was amputated, opened, and pinned out before fixation, then make 2 to 3 mm slices parallel to the endocervical canal.
- If the cervix was not amputated and pinned out before fixation, then perform radial slices at 2 to 3 mm intervals parallel to the endocervical canal.
- Tumors 2 cm or less should be entirely submitted whereas tumors larger than 2 cm can be representatively sampled.
- Tissue sections should particularly target tumor in relation to closest vaginal, paracervical/radial, and parametrial margins.
- If there is no grossly visible lesion, the entire cervix should be submitted.
- Perrpendicular sections of the vaginal margin closest to the tumor should be examined. Whether the remainder of the vaginal margin should be examined entirely en face or by representative perpendicular sections is left to local practice standards. Similarly, if there is no macroscopic tumor, the decision to examine the entire vaginal margin en face or by representative perpendicular sections is left to local practice standards.
- The parametria should be entirely submitted.
- The full thickness of the anterior and posterior walls of the corpus and of the lower uterine segment should be representatively sampled.
- The fallopian tubes should be processed using the SEE-Fim protocol and the fimbriae should be entirely submitted while the ampullary portion can be representatively sampled.
- The ovaries can be representatively sampled.

## PELVIC EXENTERATION SPECIMENS

Pelvic exenteration consists of en bloc resection of pelvic organs with the uterus and vagina. Anterior pelvic exenteration includes the urinary bladder, urethra and/or ureters. Posterior pelvic exenteration includes the rectum. Total pelvic exenteration includes both the anterior and posterior organs 51. The procedure is indicated in patients with cervical or vaginal carcinoma recurrent in the central pelvis in which conventional radiation therapy fails to control disease, and in those with advanced stage cancer that are amenable for extensive surgical resection 52,53.

### Specimen Orientation and Inking

**FIG. 9:**
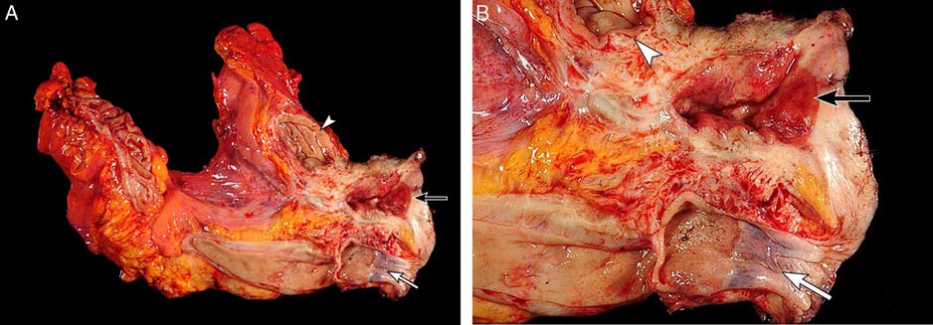
Total exenteration for recurrent cervical adenocarcinoma in the vagina (A, B, higher magnification of A). Vagina with recurrent tumor (black arrow), rectum and sigmoid (white arrow) and urinary bladder (white arrowheads).

An exenteration specimen is complex and may be difficult to orient, often due to the presence of extensive necrosis, hemorrhage and fibrosis (usually seen in the setting of previous surgery and/or radiation). The examination starts by identifying all anatomic structures (Fig. 9); close correlation with the details in the operative note and discussion with the surgeon is advised. It is useful to employ probes to identify the luminal aspect of the cervix, urethra and/or ureters. Taking photographs of the specimen is recommended to document orientation and to permit correlation with the microscopic sections.

The margins to ink are the vagina, parametria, urethra, ureters, proximal and distal rectal margins, and soft tissue margins beyond the parametria (eg, pararectal and paravesical soft tissues).

#### Recommendations

- Identify all anatomic structures present (cervix, uterine corpus, vagina, urinary bladder, rectum) in conjunction with the operative note and discussion with the surgeon.
- Margins to ink are the vagina, parametria, urethra, ureters, proximal and distal rectal margins, and soft tissue margins.

### Specimen Measurements

Measurements of all organs should be taken in the fresh state (before fixation). For the uterus and bladder, 3 dimensions should be obtained. For the rectum, vagina, and ureters, the dimensions to report include the length and the range of their diameter.

#### Recommendations

- Measure all the organs in the fresh state.

### Specimen Processing, Tumor Measurements and Tissue Sampling

Inflation of the urinary bladder and rectum with formalin and fixation of the specimen for at least several hours, or overnight, is advised to optimize the quality of the sections 15. Once fixed, the entire specimen can be hemisected to demonstrate the relationship of the tumor to the bladder, rectum and soft tissues.

The tumor should be measured in three dimensions (superior to inferior, anterior to posterior, and lateral dimensions) and its location with respect to all organs present should be reported, including whether each organ is involved or the gross distance between the lesion and the organ. Similarly, the distance of the tumor to all margins should be recorded.

Recommendations for tumor sampling are the same as those made for hysterectomy specimens. Sections should show the interface between the tumor and other structures (vagina, bladder, rectum, soft tissue). Perpendicular sections are advised to show the relationship between the tumor and mucosal surfaces of the bladder, vagina, and rectum. Representative sections of all uninvolved organs should be submitted as should any incidental lesions (eg, rectal polyps). The margins of the vagina and parametrium should be processed according to the recommendations made for hysterectomy specimens. The urethral and ureteral margins, as well as proximal and distal rectal margins, should be obtained en face. Soft tissue margins beyond the parametria (eg, pararectal and paravesical soft tissues) should be sampled perpendicular to the nearest approach of tumor. Representative en face margins can be taken if the tumor is far away.

#### Recommendations

- Inflate the urinary bladder and rectum with formalin for several hours or overnight and then hemisect the specimen.

- Measure the tumor in 3 dimensions and document its relationship to all the organs and margins.
- Representative sections of the tumor should demonstrate its relationship to all organs and margins.
- The vaginal and parametrial margins are processed as is done for a hysterectomy specimen.
- The urethral, ureteral, rectal, and soft tissue margins are processed en face, unless there is tumor nearby in which case perpendicular margins are advised.

### Intraoperative Consultation

Intraoperative consultation in the setting of a pelvic exenteration procedure is rare. It is performed to assess the closest soft tissue margins to the tumor to determine the need for additional margins. Such margins can be obtained from the main specimen or be submitted separately by the surgeon. En face sampling of the margin is more practical if the tumor is far away, but perpendicular sections are preferred if the tumor or any tissue abnormality is detected at the margin or close to it. A positive or close margin will prompt excision of additional soft tissue. The distance between tumor and margin at which re-excision is recommended has not been standardized.

A second scenario is when biopsies of the pelvic wall, abdomen and/or retroperitoneal area are taken before proceeding with the exenteration. If positive for malignancy, the exenteration is aborted. Potential pitfalls in the interpretation of these specimens include crushed artifact and radiotherapy induced changes, which can be misinterpreted as tumor.

## LYMPH NODE SPECIMENS

Pelvic lymphadenectomy is part of primary surgical treatment of all stages of cervical carcinoma except stage IA1 without lymphovascular invasion 9. Sentinel lymph node (SLN) mapping and biopsy of pelvic lymph nodes for early stage cervical cancer has emerged as a strategy to mitigate the risk for lower extremity lymphadenoma that accompanies systematic pelvic lymphadenectomy 26. SLN mapping also helps identify unusual lymph drainage patterns 54. In both Europe and the United States, current guidelines recommend consideration of SLN biopsy as an option for early stage cervical cancer 9,55. The use of intraoperative evaluation of SLN biopsy as a method to triage patients to proceed with radical surgery or to abort and pursue chemoradiotherapy has also been proposed, though diagnostic sensitivity has been shown to be a limitation 55. Prospective clinical trials evaluating the role of SLN biopsy in early stage cervical cancer management are ongoing 56,57. Para-aortic lymph node dissection is considered for stage IB1 and higher cancers 9.

Overall rates for lymph node metastasis range from 12% for HPV-associated usual type endocervical adenocarcinoma to 16.7% for HPV-independent gastric type adenocarcinoma and 22% for HPV-associated invasive stratified mucinous carcinoma 58. Among HPV-associated endocervical adenocarcinoma, the 3-tier Silva pattern of invasiveness stratifies patients into those with no risk for nodal metastasis (pattern A), ~4% risk (pattern B) and up to 25% risk (pattern C) 59. Most HPV-independent endocervical adenocarcinoma are pattern C.

The stage assignment based on lymph node involvement depends on the size of the nodal metastasis in both the 2019 FIGO staging system and the 8th edition AJCC staging system 17,60,61. In the 2019 FIGO staging, macrometastases (>2 mm) and micrometastases (>0.2–2 mm) are classified as positive lymph nodes (stage IIIC) but isolated tumor cells (up to 0.2 mm) do not affect stage. Current AJCC staging criteria classify macrometastases and micrometastases as stage pN1 and isolated tumor cells as pN0(i+) 60. Consequently, the strategy for gross management of lymph nodes should be designed to reliably detect nodal metastasis of at least 0.2 mm. The clinical significance of isolated tumor cells is still being studied.

### Distinguishing Non-SLN Versus SLN for Pathologic Processing

Specimen measurements, dissection, and tissue sampling strategies are the same for non-SLN and SLN; however, the processing of blocks is different. Therefore, at the time of gross evaluation, the pathologist should clearly determine whether the specimen is a non-SLN or SLN.

This distinction may not always be clear based on macroscopic examination of the specimen since a variety of markers are available for the surgeon to choose from to map SLN 9. Whereas direct visual mapping with blue dye may impart a blue color to the lymph node specimen, fluorescence mapping by the fluorescent marker indocyanine green or gamma probe mapping by radiocolloid technetium-99 do not affect the appearance of the lymph nodes. Thus, the pathologist should use the specimen requisition form and specimen container label to determine if a lymph node specimen is a SLN in order to determine the proper specimen management strategy.

### Specimen Measurements

The 3 dimensions of the overall lymph node specimen, including associated adipose tissue, should be documented in the gross description. If the specimen consists of multiple fragments, the dimensions of the fragments aggregated together can be reported. The number of macroscopically visible lymph nodes should be recorded as well as the dimension (long axis) of the largest node. If metastatic tumor is macroscopically visible after dissection, the largest dimension should be recorded for each involved lymph node.

#### Recommendations

- Record the size and number of macroscopically detectable lymph nodes.

### Specimen Processing and Tissue Sampling

Excess adipose tissue can be carefully trimmed from the lymph node but it should not be stripped entirely away as this may disrupt the capsule of the lymph node and may also preclude evaluation for extranodal extension of tumor if metastasis is present.

**FIG. 10:**
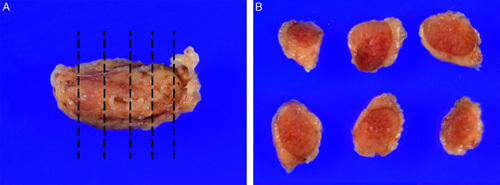
Each sentinel lymph node is sliced at 2 to 3 mm intervals perpendicular to the long axis of the node (A). All slices (B) are submitted for microscopic examination. Details of tissue block processing for ultrastaging are discussed in the text.

To maximize detection of the small volume metastasis, each lymph node should be sliced perpendicular to its long axis at intervals no more than 2 mm thick (Fig. 10). This approach has a higher chance of detecting metastasis than slicing parallel to the long axis of the node as more tissue can be evaulated 62.

If there is macroscopic metastatic tumor, a representative section can be submitted for microscopic examination. However, if there is no macroscopic evidence of metastasis, all of the sliced lymph node should be submitted. Excess adipose lacking any visible abnormality does not need to be submitted.

The number of lymph nodes in each cassette should be documented in a way that permits an accurate total count of all lymph nodes examined and of the total with metastasis.

If the specimen contains no definitive lymph nodes, the tissue should be submitted entirely for microscopic examination.

#### Recommendations

- Remove excess adipose tissue from lymph nodes (nonsentinel and sentinel) and slice perpendicular to long axis at 2 mm intervals.
- Submit all slices of each lymph node for microscopic examination unless there is an obvious macroscopic metastasis, in which case representative section is sufficient.
- Excess adipose tissue trimmed away does not need to be submitted for microscopic examination.
- Document the number of lymph nodes in each cassette to allow for an accurate total count.
- If no lymph nodes are identified grossly, submit the entire tissue for microscopic examination.

### Tissue Block Processing

For non-SLN, a single H&E-stained section per tissue block is sufficient.

For SLN, the optimal strategy for tissue block processing remains controversial. The aim to detect low-volume metastases (micrometastases and isolated tumor cells) needs to be balanced with the still unresolved questions about their clinical significance and the utilization of laboratory resources. The concept of SNL ultrastaging refers to using multiple deeper level sections of the tissue block with or without keratin immunohistochemistry aiming at the detection of low-volume nodal disease. One of the largest studies to date on SLN in cervical cancer demonstrated that up to 6.4% of metastases would go undetected if ultrastaging was not performed 63. However, the optimal parameters that define ultrastaging remain to be resolved, specifically: the number of deeper sections, the distance between the sections, and the use of keratin immunohistochemistry if all of the H&E-stained sections are negative.

Depending on the parameters of the protocol, ultrastaging can be labor and resource intensive, as well as expensive. In theory, cutting sections from the tissue block at 200 μm (0.2 mm) intervals until the block is exhausted should detect all micrometastases; this is the concept behind the strategy used in one of the ongoing prospective clinical trials that takes sections at 150  μm intervals 56. However, this means that a standard 2 mm thick slice of a lymph node would result in 10 H&E sections per tissue block. For a bilateral SLN procedure, this means at least 20 H&E sections in total (that, assuming there is only one block per side). Whether this conceptual approach is feasible outside of a clinical trial may depend on local practice conditions.

Further questions regarding keratin staining include the number of sections that should be stained and the location in the tissue block of the sections for keratin staining relative to the location of the sections for H&E staining. Thus, the evidence is clear that some form of ultrastaging is needed for SLN but there is no clear mandate on the exact details of the ideal ultrastaging protocol. Currently, the best practice should be decided at the local practice level and applied uniformly for all patients within that practice.

From a practical perspective, if SNL ultrastaging is to be performed, we recommend taking several sections at multiple intervals though the tissue block. One section should be used for H&E stain, and the others can be used for immunohistochemistry or additional H&E stains. The total number of intervals should be decided at the local practice level. Likewise, the use of routine keratin immunohistochemistry should be decided at the local practice level.

Keratin immunohistochemistry is useful not only for evaluating suspicious cells on the H&E stain, but also for confirming the classification of metastases that are at the cusp between ITC versus micrometastasis and the cusp between micrometastasis versus macrometastasis. The measurement of the size of the metastases may be more clear on a keratin stained slide than on an H&E stained slide.

#### Recommendations

- For nonsentinel nodes, a single H&E-stained section per tissue block is sufficient.
- For sentinel nodes, ultrastaging by deeper level sections should be performed; however the number of levels and the distance between levels should be decided by local practice conditions as there is insufficient evidence to make a specific recommendation.
- The role for keratin immunohistochemical evaluation of SLNs remains to be fully studied; there is insufficient evidence to make a recommendation about using keratin staining.

### Intraoperative Evaluation

European guidelines recommend that intraoperative evaluation of SLN can be used to triage whether early stage cervical cancer patients should proceed to radical surgery (if there is no nodal metastasis) or whether radical surgery should be abandoned and definitive chemoradiation pursued instead 55. This strategy is tempered by the imperfect sensitivity of intraoperative evaluation. False negative rates from 25% to 76% have been reported and while the majority of the metastases that went undetected tended to be micrometastases and isolated tumor cells, a small number of macrometastasis were also missed intraoperatively. Therefore it is recommended that intraoperative SLN evaluation be performed only if the surgeon is prepared to alter the intraoperative plan based on the results and is aware of the limitations to diagnostic sensitivity.

Intraoperatively, the SLN should be dissected using the same strategy for standard SLN processing. Remove excess adipose but avoid stripping too close to the outer surface of the node. Slice the node perpendicular to the long axis at 2 mm intervals. Evaluate each slice by frozen section but take caution not to cut too deeply into the tissue and do not perform deeper level sections except to pursue suspicious findings as that would potentially exhaust the residual tissue and impair ultrastaging of the residual tissue 64. For the permanent section processing, the standard ultrastaging protocol should be used on the remainder of the frozen section tissue block.

#### Recommendations

- Intraoperative evaluation of SLN should be performed only if the surgeon is prepared to alter the intraoperative plan based on the results and is aware of the limitations to diagnostic sensitivity
- After removing excess adipose tissue, slice the SLN perpendicular to long axis at 2 mm intervals and evaluate all slices by frozen section.
- Do not perform deeper levels intraoperatively except to pursue suspicious findings.
- Apply the standard ultrastaging protocol for permanent section processing of the remainder of the frozen tissue block.
